# Towards understanding the lifespan extension by reduced insulin signaling: bioinformatics analysis of DAF-16/FOXO direct targets in *Caenorhabditis elegans*

**DOI:** 10.18632/oncotarget.8313

**Published:** 2016-03-24

**Authors:** Yan-Hui Li, Gai-Gai Zhang

**Affiliations:** ^1^ Institute of Cardiovascular Sciences and Key Laboratory of Molecular Cardiovascular Sciences, Ministry of Education, Peking University Health Science Center, Beijing, P. R. China; ^2^ Special Medical Ward (Geratology Department), First Hospital of Tsinghua University, Beijing, P. R. China

**Keywords:** lifespan, C. elegans, insulin signaling, FOXO direct targets, bioinformatics, Gerotarget

## Abstract

DAF-16, the *C. elegans* FOXO transcription factor, is an important determinant in aging and longevity. In this work, we manually curated FOXODB http://lyh.pkmu.cn/foxodb/, a database of FOXO direct targets. It now covers 208 genes. Bioinformatics analysis on 109 DAF-16 direct targets in *C. elegans* found interesting results. (i) DAF-16 and transcription factor PQM-1 co-regulate some targets. (ii) Seventeen targets directly regulate lifespan. (iii) Four targets are involved in lifespan extension induced by dietary restriction. And (iv) DAF-16 direct targets might play global roles in lifespan regulation.

## INTRODUCTION

DAF-16, the *C. elegans* FOXO transcription factor, plays as a molecular switch in lifespan regulation [1]. When activated by reduced insulin signaling, it could extend *C. elegans*'s lifespan by activating or inhibiting its downstream genes [2, 3]. Presumably, these downstream genes largely determine how the lifespan can be extended. Yet, little is known about their positions in the regulatory network: which are directly regulated by DAF-16, and which are indirect targets.

To identify DAF-16 targets, various high throughput techniques have been used, such as microarray [2, 3], proteomics [4], and DamID (DNA adenine methyltransferase identification) [5]. Microarray and proteomics could identify DAF-16 downstream genes, but have difficult to figure out whether they are direct or indirect targets. DamID could identify DAF-16 direct targets in theory, but may have probability to identify false positives and negatives in practice [6].

Insulin signaling is remarkably conserved in *C. elegans*, *Drosophila melanogaster* and mammals, and reduced signaling of this pathway has been shown to extend lifespan in all of these animals [7]. For FOXO and its orthologs, there are different identifiers of genes, transcripts and proteins in different species. We called them all “FOXOs” hereafter. Currently, many experimentally validated FOXOs direct targets scattered in literatures [6, 8, 9]. Collecting these known targets, and then mapping them to *C. elegans* through orthologous analysis would be helpful for longevity research in *C. elegans*.

In this work, by manually reading literatures, we collected 208 experimentally validated FOXOs direct targets. Through orthologous mapping, we eventually got 109 DAF-16 direct targets in *C. elegans*. To make data easily accessible, we set up FOXODB (http://lyh.pkmu.cn/foxodb/). Bioinformatics analysis on the 109 targets revealed interesting results.

## RESULTS

### FOXODB: a database of FOXO direct targets

**Figure 1:**
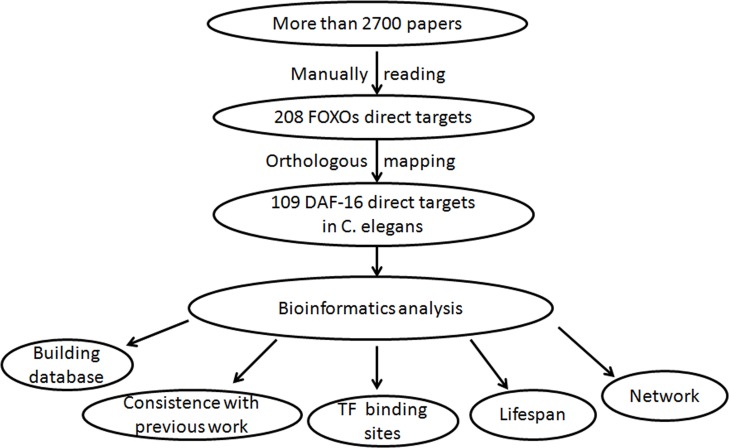
Workflow We first searched PubMed with FOXOs keywords and found more than 2700 papers. Through manually reading these papers, we got 208 FOXOs direct targets. Inparanoid was used to map FOXOs targets to their orthologs in *C. elegans*. And 109 genes were found eventually. Bioinformatics analysis on the 109 genes were done including comparison with previous results, transcription factor binding site enrichment, lifespan regulation and network topological feature analysis. And we built a database to make all data easily accessible.

As shown in Figure 1, we searched PubMed with keywords “FOXOs” and found more than 2700 papers. We manually read the papers. When a gene was determined as a FOXOs direct target, key information was extracted and record in FOXODB (http://lyh.pkmu.cn/foxodb). Rules for collecting a gene to FOXODB were strict. (i) The gene should be differentially expressed in *FOXOs* (+) *versus FOXOs* (−); (ii) FOXOs must be able to bind to the promoter of the gene; And (iii) only traditional experimental evidence(s) was adopted. Details can be found in Materials and Methods.

**Figure 2:**
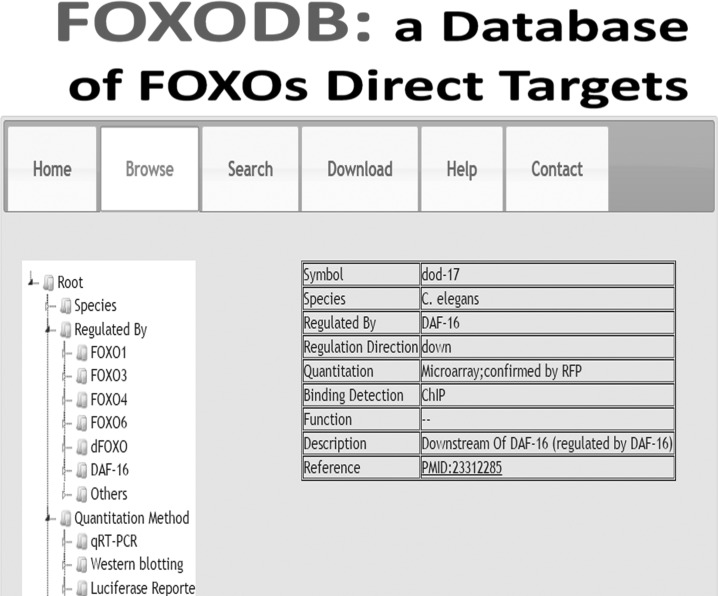
FOXODB database FOXODB is a database aiming at collecting experimentally verified direct targets of FOXOs. FOXODB is well designed and friendly to user. User could easily browse, search and download the genes.

Currently, FOXODB covers 302 entries and 208 genes, including 35, 26, and 147 direct targets in *C. elegans*, *Drosophila melanogaster* and mammals, respectively. FOXODB is well designed and friendly to user (Figure 2). As in our previous works [10, 11], FOXODB was written in PHP (Hypertext Preprocessor). We believe FOXODB will be a valuable resource to the field.

### DAF-16 direct targets significantly overlaps with previous results

Inparanoid is a database specially designed for orthologue analysis [12]. We used it to map FOXODB genes to *C. elegans* orthologs and got 109 DAF-16 direct targets eventually. (Supplementary Table 1 and 2).

The 109 targets significantly overlapped with genes found in previous works (Supplementary Table 3). For example, Murphy *et al.* found 514 differential expressed genes by comparing microarrays of *daf-16* (+) *versus daf-16* (−) [2], and 18 (2.8 in random chance, *p* = 0) of them overlapped with the 109 targets. Similar, Tepper *et al*. found 3,396 differential expressed genes [13], and 31 (18.5 in random chance, *p* = 0.0019) of them overlapped with the 109 targets. Dong *et al.* found 86 proteins differentially expressed using proteomics [4], and 12 (0.47 under random, *p* = 0) of them appeared in the 109 targets. Using DamID, 65 genes were found as potential DAF-16 targets [5], and 6 (0.31 under random, *p* = 0) of them were in the 95 targets (14 genes were excluded from the 109 targets, since they were collected from this work. 95 = 109-14). These results showed the 109 DAF-16 direct targets were reliable.

### DAF-16 and PQM-1 co-regulate some direct targets

It has been reported that DAF-16 binding element (DBE), GTAAACA or TGTTTAC, and DAF-16-associated element (DAE), TGATAAG or CTTATCA, enriched in DAF-16 regulated genes [2, 4, 13, 14]. The DBE was recognized by DAF-16, and the DAE by transcription factor PQM-1 [13]. Here, we searched both the DBE and the DAE in 1kb promoter region (relative to TSS) of the 109 targets. As a result, 30 genes contained the DBE (Supplementary Table 4), 30 genes contained the DAE (Supplementary Table 5) and 7 genes contained both (Table 1). Recent work showed that DAF-16 could even recognize DAE [15]. Taken together, these results indicated DAF-16 and PQM-1 at least co-regulate some direct targets.

**Table 1:** The seven DAF-16 direct targets that contain both DBE and DAE

GeneID	DAF-16 binding element(DBE)	DAF-16 associate element(DAE)
*daf-2*	[GTAAACA: 108]	[CTTATCA: 85]
*mtl-1*	[GTAAACA: 44]	[CTTATCA: 417]
*srp-2*	[GTAAACA: 780]	[CTTATCA: 165][TGATAAG: 448]
*rars-1*	[TGTTTAC: 925]	[CTTATCA: 697]
*T19C9.8*	[GTAAACA: 174]	[CTTATCA: 54]
*C25E10.8*	[GTAAACA: 231]	[CTTATCA: 935][TGATAAG: 267]
*daf-16*	[GTAAACA: 264]	[TGATAAG: 46]

DAF-16 binding element (DBE): GTAAACA or TGTTTAC; DAF-16 associated element (DAE): TGATAAG or CTTATCA. [GTAAACA: 108] means that GTAAACA is located 108bp relative to transcription start site.

### Seventeen DAF-16 direct targets directly regulated lifespan

GenAge [16], an useful longevity research resource, covers 681 longevity genes in *C. elegans*. Compared with the 109 targets, 17 genes overlapped, significantly higher than 3.71 under random, *p* = 0 (Table 2). Of the 17 genes, 10 were obtained by orthologous mapping. This means they were for the first time known as DAF-16 direct targets that regulate lifespan.

According to GenAge, longevity genes were classified into anti-longevity and pro-longevity [16, 17]. Knockout or suppression of anti-longevity gene, or overexpression of pro-longevity gene resulted in lifespan extension, whereas the opposite interventions led to reduction of lifespan [16, 17]. Of the 17 genes, 10 were classified as anti-longevity, 6 as pro-longevity, and one, *sod-2*, was not classified. We observed their expressions in Murphy *et al.*'s microarrays as mentioned above [2]. *gpd-2*, *pck-2* and *nnt-1* were up-regulated, and *dod-17* was down-regulated in *daf-16*(+) *vs*. *daf-16(*-). Since *gpd-2* and *pck-2* were anti-longevity, their up-regulations were not expected in *daf-16*(+) animals. Thus, we inferred that knocking out or down either *gpd-2* or *pck-2* in *daf-16*(+) might further extend lifespan. Fortunately, this inference has been validated in previous work [4]

**Table 2:** The 17 DAF-16 direct targets that directly regulate lifespan

Symbol	Longevity influence	Orthologous mapping
*din-1*	Pro-Longevity	Yes
*lgg-1*	Pro-Longevity	Yes
*mdh-1*	Pro-Longevity	No
nnt-1*	Pro-Longevity	No
*prdx-3*	Pro-Longevity	Yes
*daf-16*	Pro-Longevity	Yes
*aco-2*	Anti-Longevity	Yes
*age-1*	Anti-Longevity	Yes
*daf-2*	Anti-Longevity	Yes
*daf-7*	Anti-Longevity	Yes
*dod-17#*	Anti-Longevity	No
gpd-2*	Anti-Longevity	No
*ubh-4*	Anti-Longevity	No
pck-2*	Anti-Longevity	No
*W09D10.3*	Anti-Longevity	Yes
*lars-2*	Anti-Longevity	Yes
*sod-2*	Unclear	No

* and # represent up and down regulated in *daf-16*(+) versus *daf-16*(−), respectively. Longevity genes were classified into anti-longevity and pro-longevity. Knockout or suppression of anti-longevity gene, or overexpression of pro-longevity gene resulted in lifespan extension, whereas the opposite interventions led to reduction of lifespan. Orthologous mapping describes whether the gene was gotten from orthologous mapping (Yes) or not (NO).

### Four DAF-16 direct targets were involved in lifespan extension induced by dietary restriction

Many dietary restriction methods could extend *C. elegans*'s lifespan [18]. Some of them such as *eat* mutation or some forms of bacterial dilution do not require DAF-16, while some other forms of bacterial dilution and peptone dilution require DAF-16 [18]. Thus, it was interesting to know whether DAF-16 direct targets were involved in lifespan extension induced by dietary restriction. GenDR, a database collecting lifespan-regulating genes related to dietary restriction, covers 48 genes in *C. elegans* [19]. Here, we compared them with the 109 targets and found 4 overlapping genes: *age-1*, *hsp-12.6*, *daf-16* and *daf-2.* This was significantly higher than 0.26 under random, with *p* = 1.35E-4. This result supported that some dietary restriction methods required DAF-16 for lifespan extension.

### DAF-16 direct targets might play global roles in lifespan regulation

**Figure 3:**
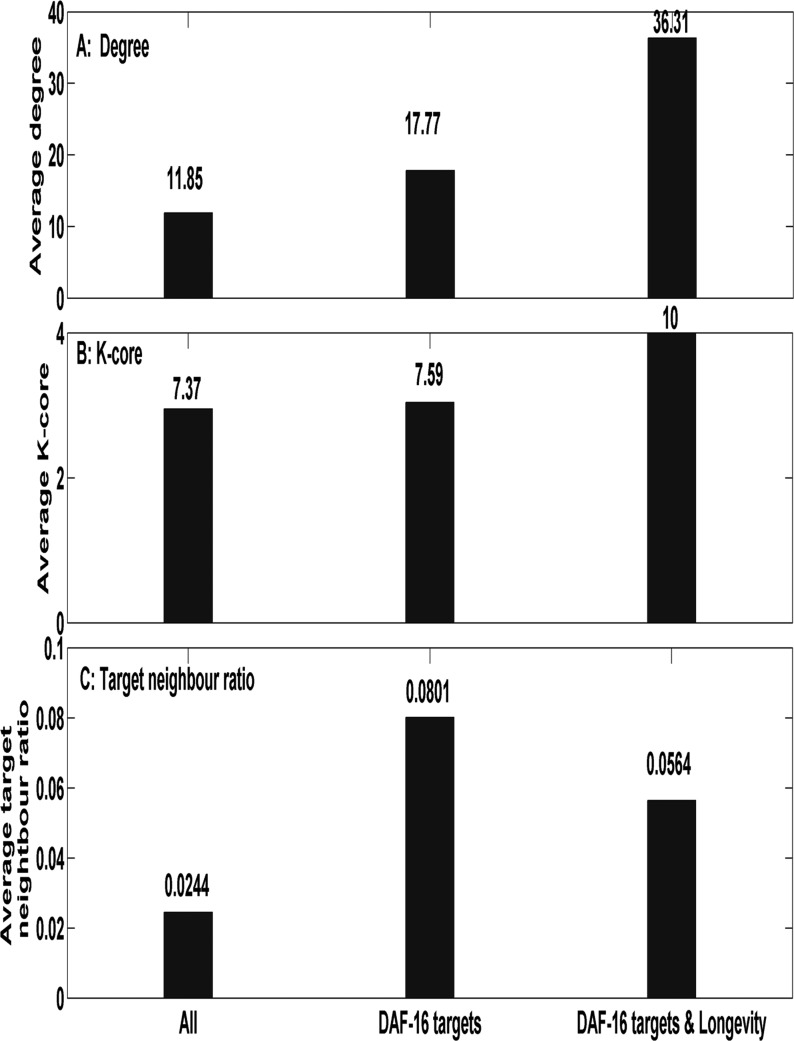
Network topological features of DAF-16 direct targets The network topological features of DAF-16 direct targets were shown. ‘All’ represents all proteins in the network. ‘DAF-16 targets’ represents the 109 DAF-16 direct targets in *C. elegans*. ‘DAF-16 targets & longevity’ represents the DAF-16 direct targets that regulate lifespan. Figure **A** is the average degree, Figure **B** is the average *K*-core, and Figure **C** is the average of target neighbor ratio.

Proteins do not function in isolation but through interaction with each other. And from network view, the more interaction partners (higher degrees) one protein has, the more important the protein might be. Here, we studied the degrees of the 109 targets, and found that the average degree is 17.77, significantly higher than 11.85, the average degree for other proteins in the network (*p* = 0.0014, Kolmogorov-Smirnov test, KS test for short). As analyzed above, 17 targets directly regulated lifespan. The average degree for them is the highest, 36.31 (see Figure 3A). This result was consistent with our previous work, the degrees of longevity genes tend to be higher than that of non- longevity genes [20].

**Table 3:** Formal representation of graph measures

Name	Function	Descriptions
Degree	*K*_*i*_	the number of interaction partners of node i
Target neighbor ratio	*K*^*p*^_*i*_	*K* ^*p*^_*i*_is the number of links between node i and proteins encoded by DAF-16 direct targets
*K*-core	*K*	A *K*-core of a graph can be obtained by recursively removing all nodes with a degree less than *K*, until all nodes in the remaining graph have a degree at least *K.*

Functions are the definitions of the topological features. Descriptions give explanations for symbols in the definitions.

*K*-core, another network index, takes into account not only the number of direct neighbors but also the placement of a protein in the network. It assumed that centrally located proteins are more important than the peripheral ones [21]. As shown in Figure 3B, the 109 targets have an average *K*-core 7.59, significantly higher than 7.37, the average for other proteins in the network (*p* = 8.4*E-4, KS test). And the 17 lifespan-regulating targets had the highest average *K*-core 10.

To know whether DAF-16 direct targets function through cooperation with each other, we computed for each protein the ‘target neighbor ratiO'. It is the ratio of the number of interaction partners that belong to the DAF-16 direct targets to its degree [22]. As shown in Figure 3C, DAF-16 direct targets tend to directly interact with each other (*p* = 2.7*E-4, KS test).

In all, these results revealed that DAF-16 direct targets tended to have more interaction neighbors, locate network center and interact with each other. This implied that DAF-16 direct targets might play global roles in lifespan regulation.

## DISCUSSION

In this work, we manually curated FOXODB by reading literatures. It now covers 208 FOXOs direct targets. To our knowledge, this is the largest. 109 DAF-16 direct targets in *C. elegans* were found by orthologous mapping. And 17 of them directly regulated lifespan. These are also important data to the field.

We searched DAF-16 binding element (DBE) in 1kb promoter region of the 109 DAF-16 direct targets, and found 30 of them contained the DBE (GTAAACA or TGTTTAC) while the others not. It was difficult to understand why so many DAF-16 direct targets did not contain DBE. For explanation, first, different works used different DBE motifs [2-4]. It was hard to know which one was correct. We chose a strict DBE motif and thus resulted in few sequence matches. If using a loose DBE motif, more genes with DBE could be found. For example, when using DBE, RTAAAYA, R = A/G, Y = C/T, as in previous work [3], 91 of the 109 targets would contain the DBE in 1kb promoter region. Second, we only searched the 1kb promoter region. Some DBE may locate outside of the region and thus not be found.

We did the first network analysis on DAF-16 direct targets. The results showed they tended to be higher in degree, locate network center and directly interact with each other. The protein interactions used for network analysis include several kinds of interactions such as physical interaction, genetic interaction and predicted interaction. However, it's worth noting that some of the interactions might be collected from literatures. Thus, the more a gene being studied, the more likely the gene has higher degree. Though the collected interactions may be only a small part of the whole data, we still cannot exclude the possibility that this might affect the results.

## MATERIALS AND METHODS

### Data source

The gene sequences were downloaded from WormBase, version 220. Protein interaction network was obtained from our previous work [20]. The network was constructed by integrating different kinds of interactions including physical interactions, genetics interactions and predicted interactions, covering a total of 7, 219 proteins and 41, 132 edges [20].

### Workflow

As shown in Figure 1, we searched PubMed with ‘FOXOs’ and found more than 2700 papers. We manually read the papers and found 208 FOXOs direct targets. Inparanoid is a database specially designed for orthologous analysis [12]. We used it to map the 208 targets to their orthologs in *C. elegans* and finally got 109 genes. Bioinformatics analysis on this list were done including comparison with previous results, transcription factor binding site enrichment, lifespan regulation and network topological feature analysis. We built a database to make all data easily accessible.

### Database creation

To collect FOXOs direct targets, we searched PubMed using keywords: ‘FOXO1, FOXO3, FOXO4, FOXO6, AFX, FKHR, dFOXO and DAF-16′. Then we manually read the papers and retrieved gene symbol, species, quantitation methods, DNA binding detection methods and function descriptions *et al.* Strict rules were used to determine whether a gene was a FOXOs direct target or not. (i) The gene should be differentially expressed in FOXOs (+) VS. FOXOs (−). The quantitation method should be traditional such as RT-PCR or western blotting. And if high throughput quantitation method such as microarray was used, the result must be further confirmed by other technique like GFP (Green Fluorescent Protein). (ii) There must be evidence showing that FOXOs could bind to the promoter region of the gene, like ChIP (chromatin immunoprecipitation) and EMSA (Electrophoretic mobility shift assay), or mutating the FOXOs binding site could significantly change the gene expression.

### Hypergeometric model

The hypergeometric model was used for calculating the significance of two gene sets with a certain number of overlapping genes. The P-value is calculated as follows:

$$P\text{ value}=1-\sum_{i=0}^{k-1}\frac{\left(\begin{array}{c} m\\ i \end{array}\right)+\left(\begin{array}{c} N-m\\ n-i \end{array}\right)}{\left(\begin{array}{c} N\\ n \end{array}\right)}$$

*N*: Number of genes in *C. elegans* genome, 20,000 was used for approximation in this work.

*m*: Number of genes in gene set 1.

*n*: Number of genes in gene set 2.

*k*: Number of overlapping genes between the *m* genes and the *n* genes.

### Kolmogorov-Smirnov test

In statistics, the two-sample Kolmogorov-Smirnov test is one of the most useful and general nonparametric methods for comparing two samples, as it is sensitive to differences in both location and shape of the empirical cumulative distribution functions of the two samples. In this work, two-sample *KS* test was used to compare the network topological features of DAF-16 direct targets and that of the remaining genes in *C. elegans*.

### Network topological features

The network topological features degree and *K*-core were computed by using an R package igraph [23]. The definitions for them can be found in Table 3.

## SUPPLEMENTARY FIGURES AND TABLES










